# Experimental taphonomy of marine algae and cyanobacteria reveals the decoupling of morphological and chemical decay patterns

**DOI:** 10.1016/j.isci.2025.113728

**Published:** 2025-10-08

**Authors:** Rut Mayo de la Iglesia, Farid Saleh, Jonathan B. Antcliffe, Pierre Gueriau, Allison C. Daley

**Affiliations:** 1 Institute of Earth Sciences, University of Lausanne, 1015 Lausanne, Switzerland; 2 Université Paris-Saclay, CNRS, Ministère de la Culture, UVSQ, MNHN, UAR3461 IPANEMA, 91192 Saint-Aubin, France

**Keywords:** Paleontology, Fossil geochemistry, Microfossil geochemistry, Aquatic biology

## Abstract

Algae are a major constituent of modern and ancient ecosystems. Investigating their evolutionary history relies on understanding their past morphologies as preserved in the fossil record. This is challenged by postmortem decay of algal cells before stabilizing as fossils. Yet, no work has documented the decay of algae in sediments under controlled experimental conditions and applied this information to interpretations of the fossil record. Here, 120 green algal samples belonging to three different morphogroups, and 40 red cyanobacteria samples were left to decay while buried in kaolinite for nine weeks. Multispectral macroimaging revealed that external morphologies were preserved long after death. Nevertheless, chemical compounds decayed over time, and pigments, such as phycoerythrin and chlorophyll became almost undetectable. Some algal morphogroups showed cell compressions, or taphonomic artifacts, which could resemble structures previously described as organelles in fossils, potentially challenging some interpretations in early eukaryotes.

## Highlights

• Algae exhibit limited external morphological decay when buried in sediments
• Algal pigments, such as chlorophyll, decay rapidly even when buried
• Some taphonomic artefacts can form inside the cell over time and may resemble organelles

## Graphical abstract

**Figure:**
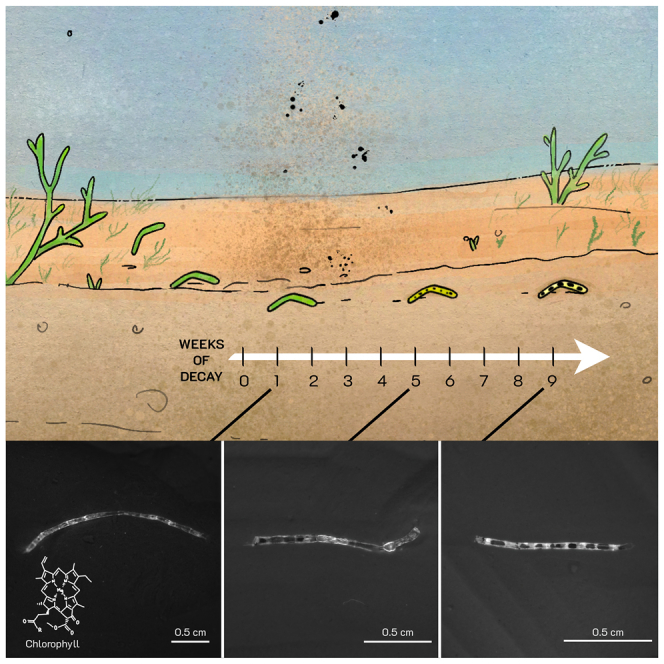

## Introduction

Algae are a polyphyletic group of photosynthetic eukaryotes that includes red, green, and brown algae, among others. Marine algae are fundamental elements of modern ecosystems, being a major group of primary producers, and are particularly dominant in coastal regions.^1^ They are an important source of oxygen for the atmosphere, contribute to the global carbon cycle, and influence sediment accumulation in shallow environments.^2^ They also offer a variety of habitats that supply animals with food, breeding spaces, and protect them from predators and other environmental processes such as wave action.^3^^,^^4^^,^^5^ During the early Palaeozoic, the function of algae could have been even greater than in the present day since shallow marine environments were more extensive, resulting in a larger surface area of habitat available to algae.^2^ Before the Mesozoic, seagrasses were lacking,^6^ therefore, primary production and carbon fixation in the seas would have primarily depended on macroalgae, as the main macrophytes at the time.^2^

The main algal groups, including non-calcified macroalgae (seaweeds), are thought to have evolved during the Neoproterozoic (1000–539 Mya).^7^^,^^8^^,^^9^^,^^10^^,^^11^ They exhibit a wide variety of shapes, such as tubiform, dichotomously branched, stoloniform, frondose, and monopodial morphologies.^2^ Although the pre-Ediacaran morphological diversity of macroalgae still needs further investigation,^12^ a notable increase in macroalgal diversity is suggested to have occurred in the early Neoproterozoic during the Tonian (1000–720 Mya).^12^^,^^13^ Later Neoproterozoic morphogroups from the Cryogenian (720–635 Mya) and Ediacaran (635–539 Mya) persisted into the Cambrian (539–485 Mya),^2^^,^^8^^,^^12^^,^^13^^,^^14^ despite the disappearance or the decline of several typical Ediacaran morphogroups by the basal Cambrian. Among these declining morphogroups are the large frondose macroalgae.^2^ Less common forms of the Ediacaran, like the monopodial morphogroup,^8^ also disappeared during the Cambrian, only to thrive again during the Ordovician.^12^

Non-calcified macroalgal fossils are often found preserved as carbonaceous compressions in fossil deposits with exceptional preservation of soft-bodied (non-mineralized) anatomy, generally referred to as Konservat-Lagerstätten.^15^ Many Konservat-Lagerstätten with Burgess Shale-type fossil algae (carbonaceous compressions in fine-grained silts and shales) are found between the latest Ediacaran and the Middle Ordovician.^16^^,^^17^^,^^18^ Although the preservation of algal fossils can be remarkably detailed, their morphologies can be altered by taphonomic processes,^8^ particularly due to decay.^19^ Despite the importance of algae in ancient ecosystems and the insights that fossil algae can provide to major evolutionary events such as eukaryogenesis,^20^^,^^21^^,^^22^^,^^23^^,^^24^ little work has been done to investigate the decay and preservation potential of algae under controlled laboratory settings. So far, experimental taphonomy has mainly been employed to investigate the decay of both animals,^25^^,^^26^^,^^27^^,^^28^^,^^29^^,^^30^^,^^31^^,^^32^^,^^33^^,^^34^^,^^35^^,^^36^^,^^37^ and cyanobacteria.^38^^,^^39^ Only one experimental study explored the taphonomy of unicellular and multicellular algae.^24^ Yet this study focused on organelle decay and did not investigate postmortem algal chemical or external morphological decay in the presence of sediments. Other studies on the decay of algae focused on the environmental effects surrounding algal bloom decomposition, such as eutrophication and release of toxins, but did not study the morphological degradation of algal cells.^40^^,^^41^

**Figure 1:**
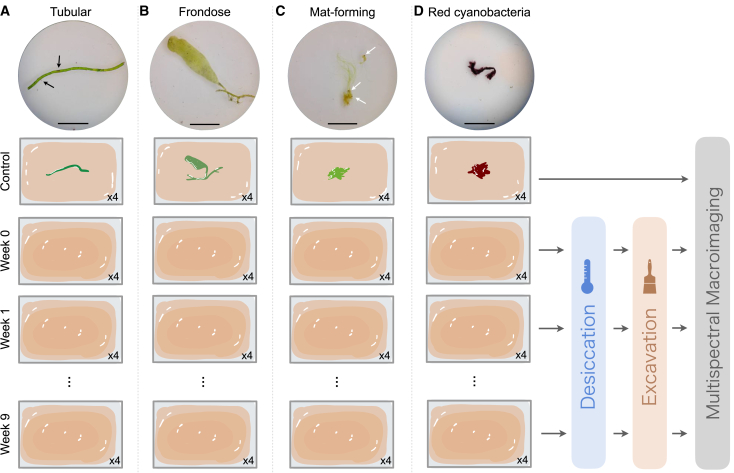
The different algal and cyanobacterial forms selected for this study, and overview of the taphonomic experimental design (A) Tubular form (*Chaetomorpha sp.*). (B) Frondose form (*Caulerpa sp.*). (C) Mat-forming green algae. (D) Mat-forming red cyanobacteria. The black arrows indicate remnants of red cyanobacteria stuck to the algae. The white arrows indicate remnants of *Artemia* cysts. Forty algal and cyanobacterial samples are placed in boxes over a 20g layer of wet kaolinite. There are four replicates per treatment per organism. The samples are buried under a 10g layer of wet kaolinite, except for the control samples, which are imaged before being buried. Week 0 samples are buried, dried, excavated and imaged on the first day of the experiment immediately after collecting the Control sample images. Every week, four replicates per species are desiccated in the oven for 4.5 h at 50°C and later excavated to reveal the buried specimens underneath. Then, they are imaged by multispectral macroimaging. This process is repeated every week for nine weeks. Scale bars: 0.5 cm.

In this study, specimens belonging to three different green algal morphogroups and one cyanobacterium (Figure S1) were left to decay while buried in clay (kaolinite) sediments. Every week for nine weeks, some samples were desiccated, excavated, and photographed using both optical microscopy and multispectral macroimaging (Figure 1). By enabling the acquisition of reflectance and luminescence images across a broad spectral range (from the ultraviolet to the near infrared), multispectral macroimaging can reveal morphological features that are invisible to the naked eye. This is particularly true for algae as their main chemical components, namely cellulose, chlorophyll, and phycoerythrin, have intense and specific luminescence signatures that allow a precise monitoring of anatomical and chemical changes throughout the decay experiment (kindly refer to the “method details” section for more information). The morphological and chemical decay of these specimens was monitored and compared to the decomposition of red cyanobacteria, which contain the same pigment as in red algae. The results show both similarities and distinctions in the decay patterns and decay rates of the various investigated organisms, providing a guide for interpreting the fossil record of algae in deep time.

## Results

### Effects of desiccation and burial

Overall, the luminescence signals for all the organisms vary significantly between controls (fresh samples before burial and desiccation; Figure S2) and Week 0 samples (buried and desiccated samples without decay) (two-way ANOVA: *F* = 116.95, *df* = 2, *p* < 0.0001) (Figure S3, Table S1). The luminescence of controls and Week 0 samples also changes significantly between the four organisms (two-way ANOVA: *F* = 21.97, *df* = 3, *p* = 0.0001; Figure S3, Table S1). The tubular and frondose macroalgae show a significant decrease in chlorophyll and cellulose after burial and desiccation in comparison to their fresh state (TukeyHSD test: *C.I. =* [0.61; 1.19], *p* < 0.0001 and *C.I. =* [0.31; 0.90], *p* < 0.0001; Figure S3). Contrastingly, neither the mat-forming algae nor the red cyanobacteria display any significant changes in their mean luminescence after desiccation and burial (Figure S3, Table S1).

### Chemical and external morphological changes over time

**Figure 2:**
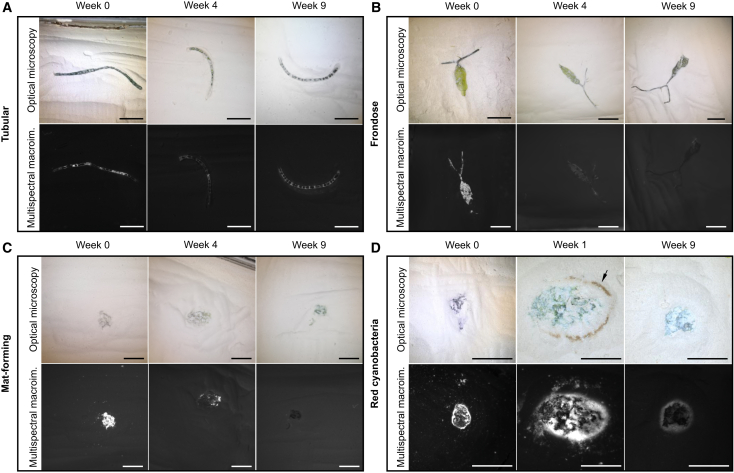
Morphological and luminescence changes of macroalgae and cyanobacteria across the nine weeks of decay (A) Tubular macroalgae at Weeks 0, 4, and 9. (B) Frondose macroalgae at Weeks 0, 4, and 9. (C) Mat-forming algae at Weeks 0, 4, and 9. (D) Red cyanobacteria at Weeks 0, 1, and 9. Top rows: images taken by optical microscopy. Bottom row: gray scale images obtained by multispectral macroimaging (macroalgae: ill. 365 nm, det. 650 ± 30 nm; cyanobacteria: ill. 525 nm, det. 650 ± 30 nm). The black arrow indicates the brown halo visible to the naked eye. See Figures S4–S7 for the gray scale images for all nine weeks of decay. Scale bars: 0.5 cm.

Despite the variable influence of burial and desiccation on the different organisms, most organisms show a decrease in luminescence over the time of the experiment except the tubular algae (Figures 2 and S4–S7). Changes in luminescence do not correlate with morphological changes as all samples retain their original external morphologies (Figures 2A–2C), except cyanobacteria samples that show a halo around the decaying organisms starting at Week 1, and which disappears over the subsequent weeks (Figure 2D).

**Figure 3:**
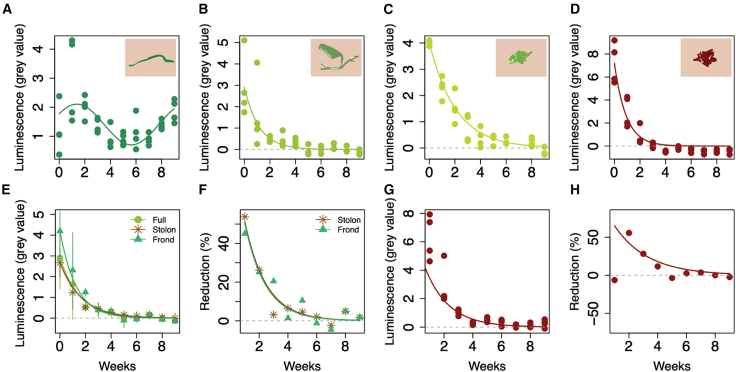
Standardized luminescence values of each algal form and cyanobacteria over time (A) Tubular form (*Chaetomorpha sp.*). (B) Frondose form (*Caulerpa sp.*). (C) Mat forming green algae. The data from Week 7 was removed due to contamination, see Figure S9 for the complete dataset. (D) Red cyanobacteria. (E) Average luminescence over time of each structure of the frondose macroalgae, including the entire alga (Full), with error bars. Data are presented as mean ± standard deviation. From Week 0–9: Full alga (2.91 ± 1.5, 1.61 ± 1.67, 0.51 ± 0.13, 0.41 ± 0.32, 0.32 ± 0.17, −0.04 ± 0.25, −0.005 ± 0.15, 0.15 ± 0.14, -4e-18 ± 0.15, −0.12 ± 0.1), frond (4.2 ± 1.54, 2.31 ± 1.81, 1.25 ± 0.18, 0.39 ± 0.34, 0.33 ± 0.21, −0.1 ± 0.34, −0.05 ± 0.15, 0.13 ± 0.19, −0.07 ± 0.12, −0.15 ± 0.11), stolon (2.67 ± 0.69, 1.23 ± 0.77, 0.53 ± 0.1, 0.45 ± 0.27, 0.27 ± 0.19, 0.15 ± 0.08, 0.1 ± 0.19, 0.16 ± 0.06, 0.03 ± 0.17, −0.01 ± 0.25). (F) Average weekly luminescence reduction for each structure of the frondose macroalgae, over time. Week 0 data (values of 0%) were removed for better visualization. (G) Standardized luminescence of the halo surrounding the red cyanobacteria samples. (H) Luminescence reduction over time of the halo surrounding the red cyanobacteria samples. The negative reduction value on Week 1 corresponds to the halo formation. The algae were illuminated at 365 nm wavelength, the cyanobacteria at 525 nm, and both luminescence signals were detected with a 650 ± 30 nm filter.

**Figure 4:**
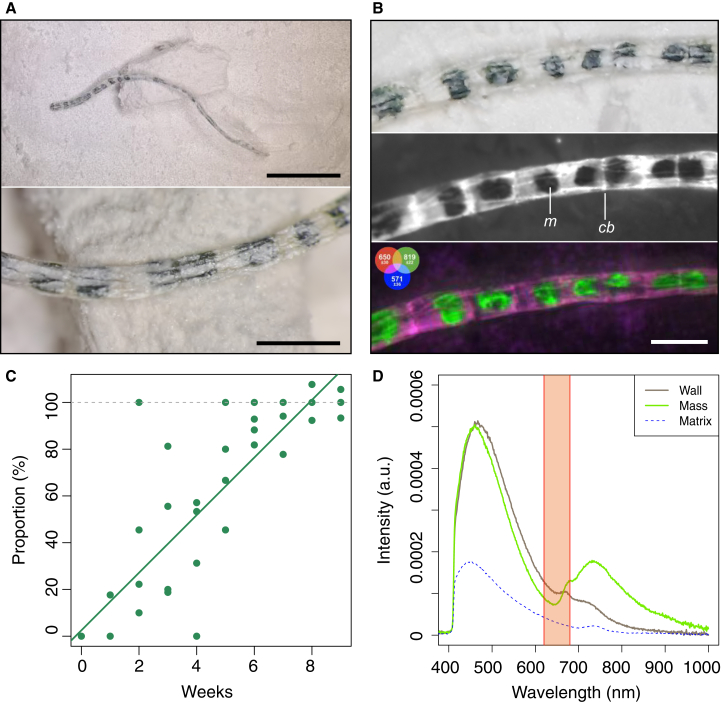
*Chaetomorpha sp.*’s internal masses after nine weeks of decay (A) Optical photographs of *Chaetomorpha sp.* showing the cells flattened by burial. Top scale bar: 0.5 cm. Bottom scale bar: 1 mm. (B) Closeup of *Chaetomorpha sp.*’s internal masses. Top: image taken by optical microscopy. Middle: gray scale images obtained by multispectral macroimaging (macroalgae: ill. 365 nm, det. 650 ± 30 nm) with labels (*m*: dark green internal masses, *cb*: cell boundary). Bottom: RGB composite image obtained by multispectral macroimaging: red (ill. 365 nm, det. 650 ± 30 nm), green (ill. 365 nm, det. 819 ± 22 nm) and blue (ill. 365 nm, det. 571 ± 36 nm). Scale bar: 1 cm. (C) Proportion of cells with internal masses per filament, over time, in *Chaetomorpha sp. Illumination at 365 nm*, *detection at 650 ± 30 nm.* (D) *UV-vis-NIR spectroscopy of the tubular macroalgal cell walls and internal masses. The region highlighted in orange represents the detection filter used in this study.*

The *Chaetomorpha* sp. tubular macroalgae samples show no significant variation in chlorophyll luminescence between Weeks 0 and 9 (Wilcoxon test: *p* = 0.3429) (Figures 2A and 3A). However, luminescence intensity does not remain constant throughout the experiment (Figure 3A). The first to seventh weeks show a significant decline in chlorophyll and cellulose (nonlinear least squares: *df* = 26, *p* < 0.0001). Then, between the seventh and ninth weeks, luminescence increases significantly (linear regression: *Adjusted R*^*2*^ = 0.74, *df* = 10, *p* = 0.0001) forming a sinusoidal pattern (Figure 3A). Nevertheless, the UV-vis-NIR emission spectra collected for the Week 9 sample (Figure 4D) strongly differ from those of Week 0 (Figure S8), particularly in the probed 650 ± 30 nm spectral domain. Notably, the cell wall exhibits an almost complete loss of chlorophyll characteristic bands and a much stronger luminescence contribution from cellulose within this spectral range, possibly explaining the sinusoidal pattern by a switch from mostly chlorophyll luminescence to mostly cellulose signal, as luminescence is predominantly emitted by the cell wall.

The frondose macroalgae (*Caulerpa* sp.), the mat-forming algae, and the red cyanobacteria show decreasing luminescence throughout the experiment (Figures 2B–2D and 3B–3D). For *Caulerpa* sp.*,* chlorophyll luminescence declined significantly (nonlinear least squares: *df* = 36, *p* < 0.0001), particularly during the first three weeks (Figure 3B). Comparison of decay in the stolon and the frond shows no significant variation between both parts (ANCOVA: *F* = 2.17, *df* = 2, *p* = 0.1182; Figure 3E). The proportion of weekly chlorophyll reductions in the stolon and the frond also show no significant difference (ANCOVA: *F* = 0.01, *df* = 1, *p* = 0.9187; Figure 3F), suggesting that chlorophyll in both frond and stolon decay at similar rates.

Chlorophyll luminescence decreases significantly for the mat-forming algae (nonlinear least squares: *df* = 32, *p* < 0.0001; Figures 2C and 3C). The Week 7 samples show a drastically lower luminescence than the other weeks (Figure S9), probably due to contamination; as an outlier, it was removed from the analysis.

A comparison between all three green algal forms shows a significant variation in chlorophyll luminescence decrease between organisms (ANCOVA: *F* = 10.46, *df* = 2, *p* < 0.0001). This is expected since chlorophyll luminescence in the tubular macroalgae shows a sinusoidal pattern (Figure 3A), Also, while it takes until Week 9 to reach zero luminescence for the mat-forming algae, the frondose macroalgae do so sooner, on Week 5 (Figures 3B and 3C). Despite this significant variation between morphogroups, when analyzing the average weekly chlorophyll reduction per morphogroup, all forms decay at a comparable speed (ANCOVA: *F* = 1.69, *df* = 2, *p* = 0.2085).

For the red mat-forming cyanobacteria, phycoerythrin luminescence declined significantly over time (nonlinear least squares: *df* = 38, *p* < 0.0001) particularly during the first three weeks of the experiment (Figure 3D). The luminescence of the halo surrounding the samples (Figure 2D) also decreased (nonlinear least squares: *df* = 34, *p* < 0.0001; Figure 3G). However, it did so at a stable rate, resulting in a non-significant weekly luminescence reduction of the halo over time (nonlinear least squares: *df* = 8, *p* = 0.547; Figure 3H). Interestingly, when comparing the average green algal luminescence reduction with that of the red cyanobacteria, the variation in decay rates is not significant (ANCOVA: *F* = 0.30, *df* = 1, *p* = 0.5858), despite phycoerythrin luminescence reaching zero around Week 3 (Figure 3D).

### Internal morphological change over time

While no major external morphological decay is observed, differences in luminescence can be accompanied by internal morphological changes resulting from the flattening of the algal cells from the pressure applied during burial. This is only true for the green tubular algae (Figure 2A), since all other organisms show no internal morphological changes (Figures 2B–2D). Within *Chaetomorpha* sp. samples, small internal masses appeared inside the cells (Figures 4A and 4B). These small masses are irregular in shape, ranging from circular to elliptical, to rectangular, with uneven and irregular margins. The masses occupy a variable percentage of the total apparent surface area of their respective cells, ranging from 23% to 71% (Figure S4). In the 650 ± 30 nm spectral domain probed by multispectral macroimaging, the cell walls are luminescent and the small, optically dark green, internal masses can be seen as black spots inside the cells (Figure 4B). UV-vis-NIR emission spectroscopy confirms that these internal masses produce very low luminescence in this spectral domain, though chlorophyll (or chlorophyll derivatives) luminescence is produced at higher wavelengths (Figure 4D) and partly fills the cells (reddish features in Figure 4B). Moreover, the spectra show that the cell wall exhibits an almost complete loss of the chlorophyll characteristic bands and a much stronger luminescence contribution from cellulose. The proportion of cells showing these internal masses on the multispectral macroimages increases significantly throughout the experiment (linear regression: Adjusted R2 = 0.73, df = 38, *p* < 0.0001), reaching 100% by the seventh week (Figure 4C).

## Discussion

### Macroalgal external morphological features are preserved long after death

Overall, all algae conserved their morphology well throughout the nine weeks of the experiment. Breakage of organisms, when it occurred, was the result of sample preparation. These results suggest that algal degradation is slower and more limited than animal decomposition. For instance, scallop muscles buried in kaolinite sediments can completely decay after only six weeks,^34^ and samples which manage to preserve that long are flattened and coated by a noticeable dark veneer in a more or less comparable pattern to what is observed on shrimp carcasses in the presence of kaolinite.^29^ Decay experiments on buried *Priapulus* worms also show internal organ shrinking and loss two days after death,^42^ although this was not the case for the cuticular anatomy, which preserves well overtime. Decay experiments on buried *Condylactis* sea anemones in organic-poor sediment^43^ show the rapid degradation of the tentacles by the first week after death. The relatively limited morphological algal decay in comparison to animals is likely due to the decay-resistant nature of the cellulose cell walls.^44^ Cellulose is well known to be a chain of glucose molecules, later assembled into microfibrils by several hydrogen bonds. This renders these microfibrils particularly rigid and insoluble. Cellulose’s role in the cell walls is mainly structural, able to withstand great mechanical stress. Therefore, it requires the combined activity of various cellulolytic and non-cellulolytic microorganisms to degrade it.^44^

### Selective internal morphological decay

External morphological preservation can nevertheless be accompanied occasionally by intracellular decay resulting in the formation of internal masses as seen in tubular algal cells (Figures 4A and 4B), which proportionally increase with time (Figure 4C). These internal masses likely result from the pressure applied during burial. The cylindrical shape of the tubular macroalgal cells, combined with the water loss during the desiccation process could have also contributed to this morphological change. A dried-out cell could facilitate the collapse of the cylindrical structure during burial since a dysfunctional vacuole would fail to maintain the cell turgor pressure.^45^ Indeed, maintaining cell turgor pressure and expanding the cell walls is one of the vacuole’s main roles,^46^ and without it, the cell cannot maintain its rigidity. A sign of rapid cell turgidity loss is also the formation of wrinkles on the surface of the body,^47^^,^^48^ which can be observed in the flattened tubular macroalgae as long folds stretching along to the tubular structure (Figures 4A and 4B).

In contrast to the tubular macroalgae, *Caulerpa* sp. do not present any clearly defined internal masses (Figure 3B), becoming barely or non-luminescent in the 650 ± 30 nm spectral domain probed by multispectral macroimaging after five weeks of decay (Figure S5). Unlike *Chaetomorpha* sp., the cell contents of *Caulerpa* sp. do not compress into internal masses after burial and desiccation (Figure 2B). The lack of cell compressions could be the result of cell wall ingrowths, which reinforce the structure of *Caulerpa* sp.^49^ These ingrowths could be an obstacle for the very large, unicellular cytoplasm, preventing it from migrating and compressing as a single internal mass. They could also stop the cell from being compressed as much as the tubular macroalgae by better distributing the mechanical compaction caused by burial.^50^ The internal morphological decay of the mat-forming algae and red cyanobacteria was not investigated at a microscopic scale, and this needs to be done in the future. However, a recent study investigated the internal decay of microscopic unicellular and multicellular algae of various ecologies in the absence of burial.^24^ They found that chloroplasts showed various deterioration signs within six weeks of decay, such as holes and fragmentation, while nuclei remained quite stable throughout their experiments.^24^ Yet, most organelles remained physically visible six weeks after death, implying that organelles within eukaryotic cells can be preserved in the fossil record.^24^ A study at this resolution could be performed in the future to compare if similar patterns of algal decay are observed in the presence of sediments.

### Extensive evidence of molecular decay

The notable decrease of chlorophyll and phycoerythrin luminescence in all samples (Figures 2A–2C and 3A–3C) indicates a pronounced chemical decay. However, one must emphasize that our experiment focused on chlorophyll and phycoerythrin only and did not monitor other pigments and biomarkers, such as hydrocarbons, which are often found in the rock record and are known as “chemical fossils”.^51^ Investigating such biomarkers in a decay context would be the next logical step to understanding how they would decay under controlled laboratory conditions, which is essential to frame their preservation over a geological time scale. Such investigations can help understand the relative abundance of major taxonomic groups and their ecology, especially in cases in which chemical fossils are preserved without evidence of body fossils.^51^^,^^52^^,^^53^^,^^54^

While most morphogroups showed similar luminescence curves, including the red cyanobacteria (Figure 2D), the tubular macroalgae did not. Rather, *Chaetomorpha* sp. exhibits a decrease in luminescence between Weeks 1 and 5, followed by an increase between Weeks 6 and 9. The initial decrease in luminescence is likely attributable to chlorophyll degradation, as is the case for other algae (Figures 3B and 3C), and as shown by UV-vis-NIR emission spectroscopy (Figure S8). The subsequent increase in luminescence between Weeks 6 and 9 is revealed by spectroscopy to result, at least in part, from enhanced cellulose luminescence, while chlorophyll bands show an almost complete loss in the cell wall after nine weeks (Figure 4D). Despite its luminescence peak at ∼450 nm, the tail of cellulose luminescence can indeed still be detected in the probed 650 ± 30 nm spectral domain (Figure 4D). This enhanced cellulose detection, however, does not by itself account for the overall increase in luminescence observed after Week 6. Rather, the increase results from a side effect of chlorophyll decay: reduced absorption by chlorophyll pigments leads to a greater probed volume of cellulose, which means that a higher quantity of cellulose contributes to the luminescence signal in each pixel. In other words, the late luminescence increase after Week 6 is not due to a reappearance of chlorophyll but likely results from an increase in both cellulose detection and probed volume following the differentiation between cell walls and the internal masses (Figure 4B).

Previous experiments highlighted that chloroplasts tend to show clear signs of deterioration after six weeks of decay,^24^ which aligns with the time frame proposed in this work, as an increase in internal masses was observed throughout the experiment. The number of cells showing these internal masses reached 100% by the seventh week after burial.

Despite the chemical degradation of algal content, decaying organic matter does not spill outside of the cells, unlike cyanobacteria (Figure 3). In cyanobacteria samples, a noticeable halo of luminescing pigments appears after one week of decay (Figure 3D) suggesting that the detected phycoerythrin in the bacteria rapidly diffuses into the surrounding environment. Yet, phycoerythrin decays rapidly both inside and outside the cyanobacteria cells, resulting in decreasing luminescence trends in both cyanobacteria and the halo (Figures 2D and 2G). This is likely due to the nature of the cyanobacterial cells. Algal cell walls are thick and rich in cellulose, while cyanobacterial cell walls are mainly composed of a peptidoglycan layer that is not as resistant to decay, allowing cell content diffusion to the surrounding environment.^38^

### Implications for the macroalgal fossil record

The observation that external algal morphology is preserved for weeks after death across different taxa when buried in kaolinite has direct implications for understanding the macroalgal fossil record. Indeed, it indicates that various algal morphologies have the potential to be faithfully preserved in the geological record when rapidly buried in kaolinite-rich sediments, as has often been interpreted to be the case in early Palaeozoic Burgess Shale-type fossils.^55^^,^^56^^,^^57^^,^^58^^,^^59^

Complex algal structures, such as stolons, often have important taxonomical roles, therefore studying their preservation potential and morphological alterations during decay is particularly helpful when interpreting the algal fossil record.^2^ Although there are major differences in the function and structure of the frond and stolon of *Caulerpa* sp., these had no impact on their decay patterns (Figure 2E) and decay rates (Figure 2F). Fronds and stolons showed similar declining luminescence curves over time (Figure 2E), implying the decomposition of complex macroalgal forms is rather homogenous. These results suggest there is no post-mortem selectivity towards a particular algal structure.

**Figure 5:**
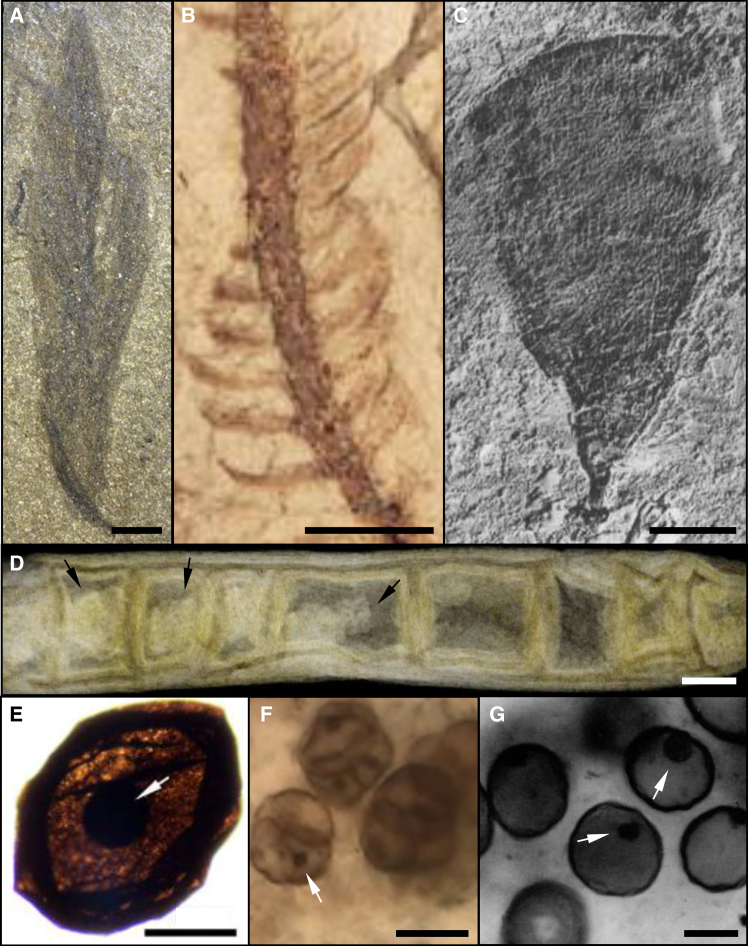
Complex macroalgal morphogroups and Precambrian fossils with internal masses (A) Frondose *Fractibeltia fibrillata* from the Kaili Formation, China.^2^ Scale bar: 2 mm. (B) *Buthograptus gundersoni*, with thallus and pinnules, from the Big Hill Formation, USA.^60^ Scale bar: 1 mm. (C) Fan-like *Flabellophyton lantianensis* from the Lantian Formation, China.^61^ Scale bar: 20 μm. (D) *Rafatazmia chitrakootensis* filament from the Tirohan Dolomite, India.^62^ Scale bar: 50 μm. (E) *Dictyosphaera* delicata cell from the Ruyang Group, China.^63^ Scale bar: 50 μm. (F) *Myxococcoides* cells from the Torridon Group, Scotland.^64^ Scale bar: 10 μm. (G) *Leiosphaeridia crassa* population from the Draken Formation, Spitsbergen.^65^ Scale bar: 25 μm. Black arrows indicate pyrenoids, white arrows indicate protoplast (E) or nucleus-like (F and G) structures. All images obtained with permission.

Exceptionally preserved frondose and stoloniform structures, which show resemblances to modern *Caulerpa* species like the one studied here, have been identified in *Fractibeltia* fossils from the Cambrian Kaili Formation, China (Figure 5A)^2^ and in *Longfengshania* fossils from the Tonian Shiwangzhuang and Jiuliqiao formations, China.^13^^,^^66^ Our results suggest that the anatomical features fossilized for these morphogroups can be accurately interpreted. Other examples of fossils presenting stolon-like structures are *Parallelphyton* fossils from the Kaili formation. These specimens have bundles of trichomes vertically attached to a thick stolon^67^ and could also be considered as examples of reliably preserved macroalgae.^2^

Our results showing high preservation potential for frondose and tubular green algae, also suggest that a good degree of preservation is likely in more complex morphogroups, such as dichotomously branched (i.e., *Miaohephyton* from the Ediacaran Miaohe Formation, China),^7^ monopodial (i.e., *Buthograptus* fossils, from the Ordovician Big Hill Formation, USA; Figure 5B),^60^ and fan-like specimens from the Ediacaran Lantian Formation, China (i.e., *Flabellophyton*; Figure 5C).^61^

### Implications for the early eukaryote fossil record

The appearance of internal masses in the *Chaetomorpha* sp. samples (Figures 4A and 4B), interpreted as decayed and compressed cellular contents, provides important insights for interpreting intracellular structures in fossils. Some Precambrian cellular fossils display centrally positioned spherical structures within cells that have occasionally been interpreted as nuclei (Figures 5D–5G). However, much debate surrounds the interpretation of subcellular structures in ancient fossils. Since the presence of a nucleus and/or organelles distinguishes eukaryotes from prokaryotes, the ability to differentiate between genuine cellular structures and taphonomic artifacts resulting from degraded and compressed cell contents is critical for tracing the evolution of early life on Earth. Our findings are not the first to demonstrate that degraded cell contents can resemble nuclei in the fossil record. Similar observations were made on degraded protoplasm in cyanobacteria,^20^^,^^68^ which bore a striking resemblance to fossilized coccoid cells from the Bitter Springs Formation, Australia (∼830 Ma), casting doubt on earlier interpretations of such features as fossilized nuclei or organelles.^69^^,^^70^ In contrast, previous decay experiments on unicellular algae suggest that nuclei should, in principle, be faithfully preserved in the geological record.^20^ Central to these debates are the size, shape, position, orientation, and consistency of subcellular structures.^71^^,^^72^^,^^73^ Eukaryotic nuclei are typically small, rarely occupying more than 20% of the cell volume,^71^^,^^72^ and tend to maintain a regular nucleus-to-cytoplasm size ratio across species.^74^ Eukaryotic nuclei are typically either spherical or oval, but the nucleus shape is known to vary depending on cell age, mutations and cell function.^71^^,^^75^ Other organelles could also be preserved, such as the pyrenoids,^62^ which have different properties from the nucleus (Kindly refer to Bengtson et al.^62^ for more details). Both organelles and taphonomic artifacts may be replicated by minerals if conditions favor authigenic mineralization, such as pyritization, phosphatization, or replacement by aluminosilicates.^76^^,^^77^^,^^78^^,^^79^^,^^80^^,^^81^^,^^82^^,^^83^^,^^84^^,^^85^^,^^86^

The irregular shape, highly variable size (relative to cell size), and indistinct margins of the internal masses in our decayed *Chaetomorpha* sp. samples set them apart from fossils in which intracellular structures are interpreted as biological in origin. For example, *Dictyosphaera* (Figure 5E) from the Paleoproterozoic Ruyang Group of China (∼1700 Mya) contains dark circular to elliptical masses with smooth margins (generally <50 μm in diameter and occupying ∼10% of the cell surface; Figure 5E). These were interpreted as biologically consolidated eukaryotic protoplasts, wherein the nucleus, surrounding cytoplasm, and plasma membrane contracted during life into a fortified mass in preparation for the resting cyst stage.^63^ Similar dark structures have been observed in exceptionally preserved unicellular organisms from the Neoproterozoic Torridon Group in Scotland (∼1000 Mya; Figure 5F)^64^ and the Neoproterozoic Draken Formation, Spitsbergen (∼750 Mya; Figure 5G).^65^ These are tiny spherical structures (generally <2 μm in the former, and <10 μm in the latter), and their size relative to the cell is consistent (typically 5%–10% of the cell surface; Figures 5F and 5G),^71^ approximating that of modern yeast nuclei (∼7% of the cell volume). Therefore, the spherical masses in these ancient fossils could plausibly represent nuclei.

In overall morphology, the internal masses in *Chaetomorpha* sp. (Figures 4A and 4B) are most comparable to *Rafatazmia* (Figure 5D), a tubular red alga with internal structures from the Mesoproterozoic Tirohan Dolomite of India (∼1600 Mya, though possibly younger^87^). In 2D images (Figure 5D), these fossils consist of cells containing a centrally positioned structure with highly irregular outlines and shapes, varying in size (generally 10%–80% of the cell area), overlapping with the size range of the structures seen in *Chaetomorpha* sp. However, in *Rafatazmia*, these irregular central structures are surrounded by additional visible matter within the cell (Figure 5D), whereas in the decayed *Chaetomorpha* sp. samples, the internal masses are isolated and lack surrounding cellular material (Figure 4A). Tomographic 3D reconstructions of the *Rafatazmia* fossils reveal that these central structures are, in fact, quite regular in shape and size, having a rhomboidal geometry consistent with identification as pyrenoids, which are dense proteinaceous bodies associated with chloroplasts and suspended in a framework of cytoplasmic matter.^62^ Even without the benefit of the 3D preservation, these biological structures could be distinguished from the decayed cellular contents in *Chaetomorpha* sp. based on the presence of other material within the cell. As such, the results of this study provide a new comparative framework that can be used when interpreting fossils, given that it is the first to experimentally observe the formation of internal taphonomy artifacts in multicellular tubular macroalgal cells while buried in sediments (Figures 4A and 4B).

### Limitations of the study

Our study aimed to determine the basic morphological and chemical decay patterns in buried algae and cyanobacteria. Because this required the desiccation and excavation of each decaying sample, a process that is irreversible, weekly observations could not be performed on the same sample. As a result, the data are not truly paired, and natural morphological and/or chemical differences between samples of the same organism type may exist. Additionally, although the excavation was carried out with great care, occasional breakages occurred in algal samples, and some material loss may have also taken place. Finally, due to time constraints, the number of samples per time point and per organism type was limited to four.

### Conclusion

Three types of green algae belonging to different morphogroups (tubular, frondose, and mat-forming) were left to decay while buried in kaolinite for nine weeks to investigate the postmortem decomposition of algae. The same experiment was carried out on mat-forming red cyanobacteria for comparison, as these cyanobacteria had a similar pigment that can be found in red algae. External and internal morphological changes were monitored, and chlorophyll and phycoerythrin decay were quantified by multispectral macroimaging for the algae and cyanobacteria, respectively. Algal external morphology remained stable throughout the experiment, likely stabilized by the robust nature of the cellulosic cell walls, meaning that fossilized algae could be interpreted with high fidelity. Despite the limited external morphological decay, all organisms showed signs of chemical decay with similar patterns of chlorophyll and phycoerythrin loss over time. Tubular macroalgae showed internal masses that superficially resemble nuclei but rather represent compressed cellular masses. These can be distinguished from nuclei based on their irregular shape and size within the cell, providing a more robust framework for interpreting structures previously described as nuclei in early eukaryote fossils. As such, the results of this work provide a unique guide to confidently interpret fossils of some of the earliest forms of life on Earth.

## Resource availability

### Lead contact

Further information and requests for resources should be directed to and will be fulfilled by Farid Saleh (farid.nassim.saleh@gmail.com).

### Materials availability

All experimental boxes are stored at the aquarium laboratory at the Institute of Earth Sciences of the University of Lausanne.

### Data and code availability

• All data necessary to replicate this work are available in the main text, the supplemental information files, and Zenodo at: https://doi.org/10.5281/zenodo.16992499.
• No code was developed for this study. All the required code to replicate this work is available on Zenodo at: https://doi.org/10.5281/zenodo.16992499.
• All other necessary material is available within the manuscript and the supplemental information files.

## Acknowledgments

Mathieu Thoury (IPANEMA) designed the multispectral macroimaging approach and provided access to the UV-vis-NIR spectroradiometer. Claudia Baumgartner is thanked for her assistance during the optical microscopy observations. Antoine Vite is thanked for fruitful discussions. R.M.d.l.I. and F.S.’s work is supported by an SNF Ambizione grant (PZ00P2_209102), and the Institute of Earth Sciences of the University of Lausanne. J.B.A.’s work is supported by an SNF Synergia grant (198691) awarded to A.C.D. and three other PIs.

## Author contributions

R.M.d.l.I., F.S., and A.C.D. planned the experiment with the help of J.B.A. R.M.d.l.I. carried out the experiment with the help of F.S. and J.B.A. R.M.d.l.I. collected data with the help of P.G. P.G. developed the multispectral macroimaging setup and conducted and interpreted the UV-vis-NIR spectroscopy data. R.M.d.l.I. interpreted and discussed the rest of the results with the help of all co-authors. R.M.d.l.I. wrote the manuscript and made the figures with the help of all co-authors.

## Declaration of interests

The authors declare no competing interests.

## Declaration of generative AI and AI-assisted technologies in the writing process

During the preparation of this work, some authors used ChatGPT in order to correct grammatical errors in the text. After using this tool/service, the authors reviewed and edited the content as needed and take full responsibility for the content of the publication.

## STAR★Methods

### Key resources table

**Table:** 

REAGENT or RESOURCE	SOURCE	IDENTIFIER
**Biological samples**		

Parts of green macroalgae	Aquarium Laboratory at the Institute of Earth Sciences, University of Lausanne	N/A
Droplets of green mat-forming algae	Aquarium Laboratory at the Institute of Earth Sciences, University of Lausanne	N/A
Droplets of red cyanobacteria	Aquarium Laboratory at the Institute of Earth Sciences, University of Lausanne	N/A

**Deposited data**		

Optical photographs	This paper	https://doi.org/10.5281/zenodo.16992499
Multispectral macroimaging photographs	This paper	https://doi.org/10.5281/zenodo.16992499
Plots	This paper	https://doi.org/10.5281/zenodo.16992499
Code	This paper	https://doi.org/10.5281/zenodo.16992499
Raw tables	This paper	https://doi.org/10.5281/zenodo.16992499

**Experimental models: Organisms/strains**		

*Chaetomorpha* sp.	Aquarium Laboratory at the Institute of Earth Sciences, University of Lausanne	N/A
*Caulerpa* sp.	Aquarium Laboratory at the Institute of Earth Sciences, University of Lausanne	N/A
Unidentified green algae	Aquarium Laboratory at the Institute of Earth Sciences, University of Lausanne	N/A
Unidentified cyanobacteria	Aquarium Laboratory at the Institute of Earth Sciences, University of Lausanne	N/A

**Software and algorithms**		

R Studio	https://www.r-project.org/	N/A
ImageJ	https://imagej.net/ij/	N/A

**Other**		

Kaolinite clays	ZF Chemstore	N/A

### Experimental model and subject details

Green seaweed species with tubular (*Chaetomorpha* sp.) and frondose (*Caulerpa* sp.) morphologies, one mat-forming green alga (various species, Figure S1), and one mat-forming red cyanobacterium species were selected for this study (Figure 1). The algal organisms were chosen for their contrasting morphological characters, ease of culturing in large quantities in marine aquaria, well-known anatomy, and their similarity to Ordovician, Cambrian, and Precambrian macroalgal fossils^12^. The cyanobacteria were chosen because they have the same sulphur-rich pigment, phycoerythrin as in red algae. All organisms were grown in saltwater (salinity: 1.021–1.025 psμ) aquariums, at room temperature (21.5°C) in the presence of marine shrimps, *Limulus*, and *Artemia* cysts (Figure 1).

*Chaetomorpha* sp. (Figure 1A) is a free-floating intertidal and supralittoral green seaweed^88^ growing in twisted tubular filaments. Its cylindrical cells are around 0.8 mm long and 0.4 mm wide. The overall pigmentation of this species is bright green with tiny red spots. This species grows abundantly when cultivated in marine aquaria. In total, forty samples were chosen and cut between 1.5 and 2.5 cm in length each. *Caulerpa* sp. (Figure 1B) is a small green seaweed formed by a long creeping stolon with rhizoids, and several flat, upright fronds are generally between 0.5 and 1.5 cm long. Each individual consists of one single multinucleated cell and is characterized by the presence of cell wall ingrowths (trabeculae) with structural functions.^49^ While the fronds are photosynthetic, the stolon and rhizoids can have tethering and nutrient uptake functions.^49^ Forty samples were cut to contain one frond with a piece of a stolon attached.

The mat-forming green algae (Figure 1C) and marine red cyanobacteria (commonly called “red-slime algae”) (Figure 1D) used for this study were composed of microscopic filaments covering all surfaces in the aquariums, including the other studied seaweeds. However, whenever this was the case, the studied seaweeds were excessively rinsed by reverse osmosis water to remove the remains of the mat-forming green algae attached to their surface. The mat-forming green alga and red cyanobacteria samples were then observed using optical microscopy (Zeiss AX10 Imager.M2m) with an Axiocam 506 Color connected camera and the ZEN (version 2.5) imaging software. The mat-forming green alga samples were formed of a high variety of uni- and multi-cellular green algal taxa (Figures S1A and S1B). A minimum of five different morphotypes and taxa were present in each sample, including filamentous taxa, desmids and diatoms. Although not green algae, diatoms represented only a minor fraction of each sample; for the sake of simplicity, the term “mat-forming green algae” is used for these samples throughout the manuscript. Red cyanobacteria, nematodes, arthropods, and zooplankton contamination were also observed in some of these samples (Figures S1C–S1F). Small amounts of green algal specimens were present in the red cyanobacteria samples, but these are easily recognizable due to their green color and were avoided as much as possible (Figure S1G). Forty samples of each of the mat-forming green algae and red cyanobacteria were used in the experiment. The latter consisted of 0.5 cm-wide droplets.

### Method details

#### Experimental decomposition setup

The organisms studied were buried in wet sediments to simulate rapid burial, as it is known to increase preservation by limiting the deterioration of the carcasses by environmental factors, such as scavengers and oxygen.^89^ The sediment used for burial consisted of the clay mineral kaolinite ([Al_2_Si_2_O_5_(OH)_4_], ZF Chemstore), as it has been associated with better carcass preservation in the rock record and modern experiments.^55^^,^^56^^,^^57^^,^^90^ Kaolinite, in powder form, was mixed with 68% of its weight in saltwater to obtain a muddy consistency. Distilled water and artificial sea salt (Red Sea) were mixed to prepare saltwater with a 1.025 psμ salinity measured by a digital salinity meter (SM01 Sakura Aquatics).

The forty samples per organism were deposited individually inside rectangular (4.5 × 6 × 2 cm^3^) lidded plastic boxes containing each 20 g of wet kaolinite and covered with a 10 g layer of wet kaolinite (Figure 1). The organisms were left to die and decompose for a total of nine weeks at room temperature (21.5°C) in a dark chamber with no light. Plastic boxes were not sealed, and oxygen likely diffused inside the experimental box. Every week, four replicates per species were removed from the experiment and left to dry in an oven (Binder EDS056-120V) at 50°C for 4.5 h and then were excavated by delicately scraping the sediments away with a small steel spatula and a brush. Excavation stopped once the upper surface of the sample was fully exposed, but the sample was still attached to the underlying sediments. In rare instances, the cell walls broke during preparation; however, these occurrences were documented to ensure that any preparation artifacts do not bias subsequent interpretations. After excavation, the weekly set of samples was imaged using an Olympus SZX10 microscope and an Olympus SC50 connected camera running the Olympus Stream Basic (2008–2016) software. Then they were imaged using multispectral macroimaging (details below).

To control for the effects of desiccation and burial, four samples of each organism were imaged before being buried under the 10g kaolinite layer; and are termed as “Controls” thereafter (Figure 1). These represent the fresh state of the organisms. The samples termed “Week 0” are the control samples after they were set, dried, excavated, and imaged, all on the same day (Figure 1).

#### Photoluminescence imaging and spectroscopy

We used multispectral photoluminescence (often referred to by the less inclusive term ‘fluorescence’; see Toussaint et al.^91^ for terminological clarifications) macroimaging to highlight anatomical and compositional contrasts that are not discernible through classical optical observation. This approach follows the same principle as multispectral fluorescence microscopy used in biology and medicine,^92^^,^^93^ collecting luminescence images across different spectral ranges, from ultraviolet (UV) to near-infrared (NIR), as various chemical and structural compounds respond to different wavelengths. However, in the case of ‘macroimaging’, it operates at the macro-rather than micro-scale, i.e., over an imaged field of view spanning several centimetres. The setup used in this study consists of a low-noise 2.58 megapixel sCMOS camera with sensitivity from 200 to 1000 nm, fitted with a UV-VIS-IR 60 nm 1:4 Apo Macro lens (CoastalOptics), and mounted on a camera stand. A detection filter wheel is positioned in front of it, with eight interference band-pass filters (Semrock) to collect images in eight spectral ranges selected to cover most of the 415–1020 nm domain. The illumination source was 16 LED lights ranging from 365 to 770 nm wavelength (CoolLED pE-4000), coupled to a liquid light guide fitted with a fiber optic ring light guide. As such, more than 90 different illumination/detection couples are available.^94^^,^^95^^,^^96^

The illumination/detection couples used for this study were chosen based on the best signals detected during preliminary observations on fresh algae and cyanobacteria. Two couples were selected in this study, and the chemical components luminescing under these illumination/detection couples were identified using the literature. The first couple [illumination: 365 nm (UV); detection: 650 ± 30 nm (red)] corresponds to the emission of chlorophyll pigments.^97^ . This identification was confirmed for the tubular algae by UV−vis−NIR emission spectroscopy measured using a Specbos 1211UV spectroradiometer (JETI) (Figure S8). UV illumination was provided by a U1c 6W 365 nm UV LED flashlight (JAXMAN), and a long-pass filter (cutoff wavelength: 410 nm) was placed in front of the detector to remove diffuse reflection of the excitation by the sample. Spectra (up to 1000 nm) were collected from ∼1 mm^2^ area pinpointed using the spectroradiometer’s internal target spot laser.

Chlorophyll pigments are located inside the photosystem protein complexes on the thylakoid membranes inside algal and plant chloroplasts. They play a crucial role as light harvesters and in electron transfer during photosynthesis.^98^ The tail of the emission from the cellulose composing the algal cell walls may also be detected with this couple (Figure S8),^99^ although at a much lower intensity. Therefore, the photoluminescence intensity measured in the red domain when exited by UV represents both chlorophyll and to a lesser extent cellulose.

The second couple [illumination: 525 nm (green); detection: 650 ± 30 nm (red)] corresponds to the emission of phycoerythrin pigments.^100^ These red pigments are found in the phycobilisome complexes of the photosystem II red algal chloroplasts and in cyanobacteria.^101^ These pigments play an important role as complementary light-harvesting molecules, when in a low-light environment.^102^

Three additional luminescence images were collected for the tubular algae at Week 9 in the 571 ± 36 nm (yellow), 650 ± 30 nm (red) and 819 ± 22 nm (NIR) domains under 365 nm (UV) illumination to produce an RGB composite image.

#### Image collection and processing

Multispectral images were collected using the Micro-Manager software (2.0.0-gamma1) setting the CCD temperature to −20°C and the Clear mode to “pre-sequence”. They are 16-bit greyscale images, with pixels appearing white where the luminescence signal of the sample is highest, and black where it is lowest. Therefore, the gray value of each pixel can be used as a proxy for luminescent matter concentration. The average gray value of the algal and cyanobacterial samples was extracted from the surface area within a drawn outline of the organism (Figure S10). For the frondose macroalgae, the average gray value was also measured for the frond and the stolon separately. A homogeneous spot of the background was measured for each sample, which was then subtracted from the organism’s average gray value, and then the total was divided by the exposure time (since exposure time varied between images). This was done to standardize the luminescence measurements for all samples. For the red cyanobacteria, which soon displayed a halo of luminescence around the sample, the average gray value of this halo was also measured by selecting only the outline of the halo, avoiding the sample.

The RGB composite image was generated by stacking the greyscale images for the three detection/illumination couples and attributing them red, green and blue color scales (dark to bright pixels corresponding to lowest and highest luminescence, respectively): red for emission at 719 ± 30 nm (NIR), green for emission at 571 ± 36 nm (yellow), and blue for emission at 650 ± 30 nm (red), all under under 385 nm (UV) illumination. All image processing, luminescence and area measurements were done using the software ImageJ (version 1.53).

### Quantification and statistical analyses

To test for the effects of desiccation and burial, Week 0 samples (dried and prepared) were compared to the Controls (fresh samples before their burial, desiccation, and preparation) using a two-way analysis of variance (ANOVA). The fixed effect variable used was the luminescence and the random effect variables were the treatment (Control and Week 0) and organism type (tubular macroalgae, frondose macroalgae, mat-forming algae and red cyanobacteria). The interaction between both variables (treatment × organism) was also analyzed. The two-way ANOVA was chosen since both the treatments and the organism types are fixed factors, and only the luminescence variable is continuous. These analyses tested whether (a) the luminescence varied depending on the organism; (b) the luminescence varied after burial and desiccation; and (c) burial and desiccation affected the luminescence differently for each organism. The Tukey Honest Significant Difference test (TukeyHSD), which compares the means of the levels of a factor, was later performed on the same data. This test identified which treatment-organism pair showed significantly different mean luminescence values. To ensure the normality of residuals and homogeneity of variances, the luminescence measures were transformed by 1.4^th^ root. This transformation was set after verification with a Shapiro-Wilk normality test, and the best result (here, *W* = 0.91, *p* = 0.002) was selected.

The evolution of luminescence over time for each organism was analyzed by non-linear regressions using the least square estimation method. The random variable, time, was fit into the following exponential function: *y = a·e* ^*(r·x)*^ where *a* is the standardized luminescence value at Week 0 (*t=0*), and *r* is the growth rate (in this case, the decay rate will be negative, as organic material is lost with time). Whenever decay did not proceed as an exponential function, linear regressions were used instead, using time as a random variable. The standardized luminescence measures were transformed by adding the difference between the largest negative value and zero, to avoid negative values, and then adding 0.01, to avoid null values.

For tubular macroalgae, the difference in luminescence between the first and last weeks of treatment was also tested with a Wilcoxon test for unpaired data. It is worth noting that for tubular algae, cell compressions (or internal masses) started to appear over time. The average size of these internal masses proportional to the total size of the cell was calculated based on five cell measurements per sample.

The luminosity difference in the decay pathway between the stolon and the frond of *Caulerpa* sp. was analyzed with an analysis of covariance (ANCOVA). The standardized luminescence was used as the fixed effect variable, and the time and the structure type (frond and stolon) as random variables. The interaction between both (time × structure) was also analyzed. An ANCOVA was chosen since the time variable is continuous while the structure type is a fixed factor. This analysis tested whether (a) the luminescence varied depending on the structure type; (b) the luminescence varied over time; and (c) the evolution of luminescence over time was different between both structures. To ensure the normality of residuals and correct the highly triangular distribution of residual variances, the standardized luminescence measures were transformed by natural logarithm.

Proportions of weekly luminescence reduction were calculated. This was done for each organism and both the frond and stolon of *Caulerpa* sp. For the calculation, the average standardized luminescence values (L) of each week (t_*i*_) were used as follows:

Weekly luminescence reduction = [(L(*t*_*i*_) – L(*t*_*i+1*_)) ÷ L(*t*_*0*_)] · 100.

To analyze the difference in decay rates between all organisms, but also between *Caulerpa* sp.’s structures, the proportions of luminescence reduction were analyzed using an ANCOVA. The proportions were set as fixed variables, with time and organism type (or structure) as random variables. Their interactions (time × organism; time × structure) were also analyzed. The standardized luminescence and proportions were also transformed by natural logarithm, since their residual variances showed highly triangular distributions, and were not homogenous.

The surface area of the internal masses (*N* = 20) that appeared over time in *Chaetomorpha* sp., relative to the apparent surface area of the corresponding cells, was also calculated using ImageJ (version 1.53).

All statistical analyses were carried out using R 4.2.1 (R Core Team 2022). The functions *cor.test*, *anova*, *lm*, *nls*, *aov* and *TukeyHSD* from the package *stats* were used to perform the linear regressions, analyses of variance, and covariance.
