# Supplementary material for: Experimental taphonomy of marine algae and cyanobacteria reveals the decoupling of morphological and chemical decay patterns
Source: iScience. 2025 Oct 8;28(11):113728. doi: 10.1016/j.isci.2025.113728 (PMC12597004; doi:10.1016/j.isci.2025.113728)
Supplement: Document S1. Figures S1–S10 and Table S1 [file mmc1.pdf]

iScience, Volume *28*

Supplemental information

Experimental taphonomy of marine algae and cyanobacteria reveals the decoupling of morphological and chemical decay patterns

Rut Mayo de la Iglesia, Farid Saleh, Jonathan B. Antcliffe, Pierre Gueriau, and Allison C. Daley

Supporting Information

Table S1: Results of the TukeyHSD test to identify which pairs of treatment-organism (tubular macroalgae, frondose macroalgae, mat-forming algae and red cyanobacteria) show significantly different mean luminescence values. Significant differences are highlighted in bold.

	Difference	Confidence intervals		*p-value*
		Lower	Upper	
Control vs Week 0	0.48	0.39	0.57	**<0.0001**
Mat-forming vs Frondose	-0.25	-0.43	-0.08	**<0.0001**
Cyanobacteria vs Frondose	0.00	-0.17	0.17	1.0000
Tubular vs Frondose	-0.42	-0.59	-0.25	**<0.0001**
Cyanobacteria vs Mat-forming	0.26	0.08	0.43	**<0.0001**
Tubular vs Mat-forming	-0.17	-0.34	0.01	0.0700
Tubular vs Cyanobacteria	-0.42	-0.59	-0.25	**<0.0001**
[Control : frondose] vs [Week 0 : frondose]	0.90	0.61	1.20	**<0.0001**
[Week 0 : mat-forming] vs [Week 0 : frondose]	0.13	-0.19	0.44	0.8700
[Control : mat-forming] vs [Week 0 : frondose]	0.31	0.02	0.60	**0.0300**
[Week 0 : cyanobacteria] vs [Week 0 : frondose]	0.34	0.05	0.64	**0.0100**
[Control : cyanobacteria] vs [Week 0 : frondose]	0.56	0.27	0.85	**<0.0001**
[Week 0 : tubular] vs [Week 0 : frondose]	-0.27	-0.57	0.02	**0.0800**
[Control : tubular] vs [Week 0 : frondose]	0.34	0.04	0.63	**0.0200**
[Week 0 : mat-forming] vs [Control : frondose]	-0.78	-1.09	-0.46	**<0.0001**
[Control : mat-forming] vs [Control : frondose]	-0.60	-0.89	-0.30	**<0.0001**
[Week 0 : cyanobacteria] vs [Control : frondose]	-0.56	-0.85	-0.27	**<0.0001**
[Control : cyanobacteria] vs [Control : frondose]	-0.34	-0.64	-0.05	**0.0100**
[Week 0 : tubular] vs [Control : frondose]	-1.18	-1.47	-0.89	**<0.0001**
[Control : tubular] vs [Control : frondose]	-0.57	-0.86	-0.28	**<0.0001**
[Control : mat-forming] vs [Week 0 : mat-forming]	0.18	-0.14	0.50	0.5700
[Week 0 : cyanobacteria] vs [Week 0 : mat-forming]	0.22	-0.10	0.53	0.3500
[Control : cyanobacteria] vs [Week 0 : mat-forming]	0.43	0.12	0.75	**<0.0001**
[Week 0 : tubular] vs [Week 0 : mat-forming]	-0.40	-0.72	-0.09	**0.0100**
[Control : tubular] vs [Week 0 : mat-forming]	0.21	-0.11	0.52	0.4000
[Week 0 : cyanobacteria] vs [Control : mat-forming]	0.04	-0.26	0.33	1.0000
[Control : cyanobacteria] vs [Control : mat-forming]	0.25	-0.04	0.54	0.1300
[Week 0 : tubular] vs [Control : mat-forming]	-0.58	-0.87	-0.29	**<0.0001**
[Control : tubular] vs [Control : mat-forming]	0.03	-0.26	0.32	1.0000
[Control : cyanobacteria] vs [Week 0 : cyanobacteria]	0.22	-0.08	0.51	0.2600
[Week 0 : tubular] vs [Week 0 : cyanobacteria]	-0.62	-0.91	-0.33	**<0.0001**
[Control : tubular] vs [Week 0 : cyanobacteria]	-0.01	-0.30	0.28	1.0000

[Week 0 : tubular] vs [Control : cyanobacteria]	-0.83	-1.13	-0.54	**<0.0001**
[Control : tubular] vs [Control : cyanobacteria]	-0.22	-0.52	0.07	0.2200
[Control : tubular] vs [Week 0 : tubular]	0.61	0.32	0.90	**<0.0001**

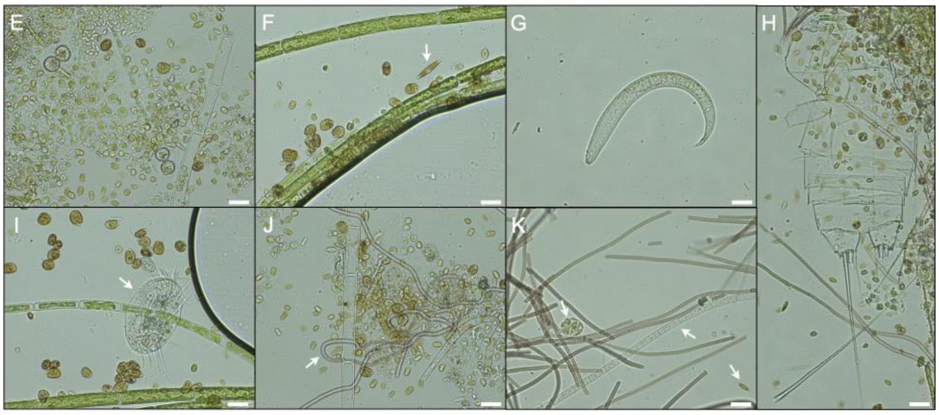

Figure S1: Observations of the green mat-forming algae and cyanobacteria by optical microscopy. (A) Filamentous algae and desmids. (B) Filamentous algae, desmids, and diatoms (white arrow). (C) Nematode. (D) Copepod. (E) Euplote (white arrow). (F) Red cyanobacteria contamination (white arrow). (G) Green alga contamination of red cyanobacteria (white arrows). Scale bars: 20 µm.

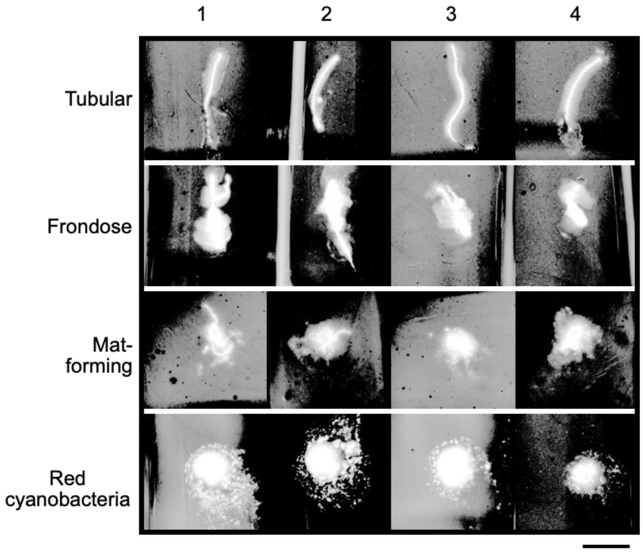

Figure S2: Grey scale images presented at a logarithmic scale of all four algal and cyanobacterial forms before burial and desiccation (Control samples). Images obtained by multispectral macroimaging. Algae: illumination 365 nm, detection 650±30 nm. Red cyanobacteria: illumination 525 nm, detection 650±30 nm). Scale bar: 1 cm.

Formatted: Justified

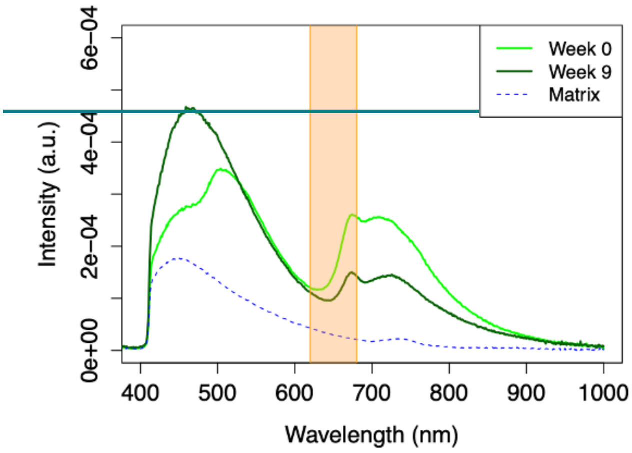

~~**Figure S2**: UV–vis–NIR spectroscopy of the tubular macroalgae, after 0 and 9 weeks of decay. Luminescence bands in the 650–800 nm domain are characteristic of chlorophyll. No chlorophyll luminescence is observed for the kaolinite sediment, with the luminescence centred around ~450 nm originating from cellulosic 'dust' residue on the sample surface (note that the band at ~730 nm is the second harmonic of the 365 nm illumination, visible here due to the extremely low overall luminescence signal produced by only a few 'dust' residues). The orange selection represents the 650 ±30nm detection filter used in this study.~~

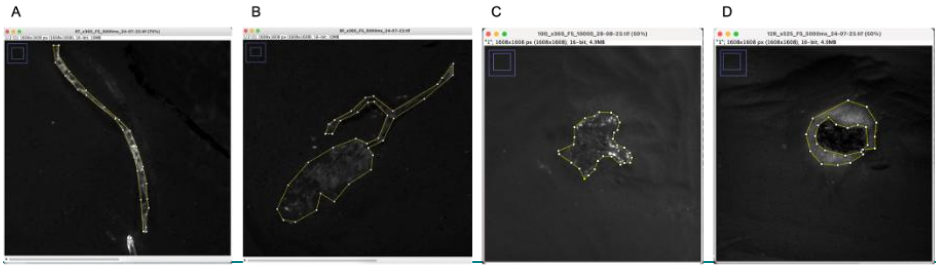

~~**Figure S3: Examples of outline selection for each organism.** (A) Tubular macroalga sample after 3 weeks of decay. Here the entire sample is shown outlined for measurement. (B) Frondose macroalga sample after 3 weeks of decay. Here the entire sample is shown outlined for measurement. In addition, both the frond and the stolon were outlined for separate measurements. (C) Mat-forming algae sample after 3 weeks of decay. Here the entire sample is shown outlined for measurement. (D) Red~~

~~cyanobacteria sample after 3 weeks of decay. Here the luminescence halo is shown outlined for measurement. In addition, the sample alone was outlined for measurement.~~

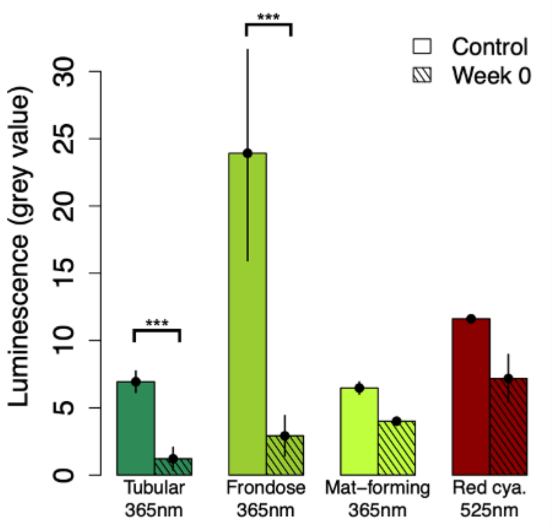

Figure S34: Average luminescence values of the algal and cyanobacterial samples, controlling for the effects of desiccation and burial, with error bars. The Control samples were measured before burial and desiccation. The Week 0 samples were measured after burial, desiccation and preparation. Both samples were prepared and measured on the same day, thus without decay. Data are presented as mean ± standard deviation: Tubular (Control = 6.93±0.78 ; Week 0 = 1.21±0.84), Frondose (Control = 23.91±7.98 ; Week 0 = 2.91±1.50), Mat-forming (Control = 6.47±0.45; Week 0 = 4±0.11) and Red cyanobacteria (Control = 11.6±0.15 ; Week 0 = 7.18±1.77). Black bars and asterisks indicate the significant combinations from the Tukey HSD Test (* p>.01; ** p<.001; *** p<.0001). All sample sizes equal four, except the Week 0 mat-forming algae, which equals three. The illumination wavelength is noted in nanometres for each sample on the x-axis. Luminescence was detected with a 650±30 nm filter.

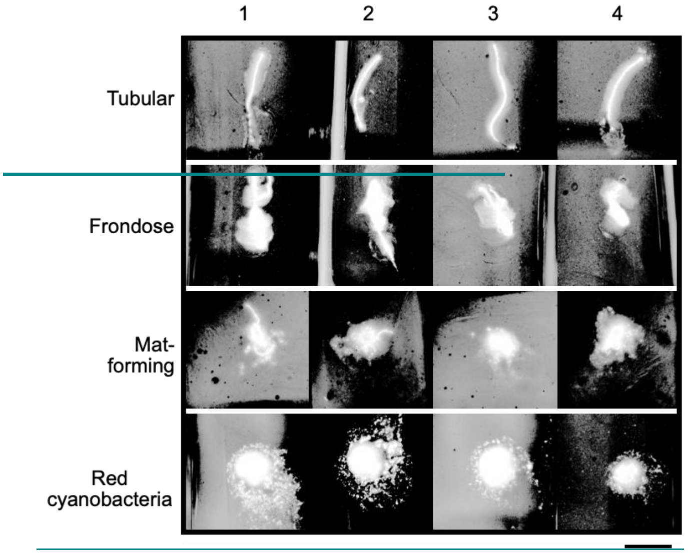

Figure S5: Grey scale images presented at a logarithmic scale of all four algal and cyanobacterial forms before burial and desiccation (Control samples). Images obtained by multispectral macroimaging. Algae: illumination 365 nm, detection 650±30 nm. Red cyanobacteria: illumination 525 nm, detection 650±30 nm). Scale bar: 1 cm.

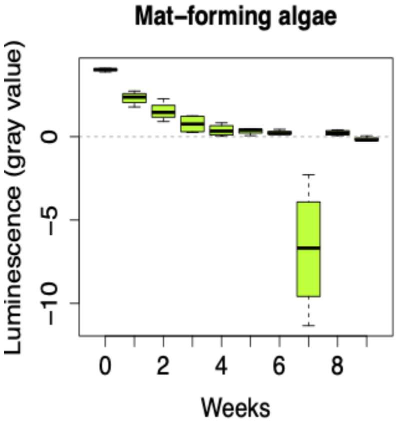

Figure S6: Standardized luminescence values of the mat-forming green algae, over time. Including the Week 7 data removed from the main manuscript, as it is considered an outlier. Illumination at 365 nm wavelength and luminescence detected with a 650±30 nm filter.

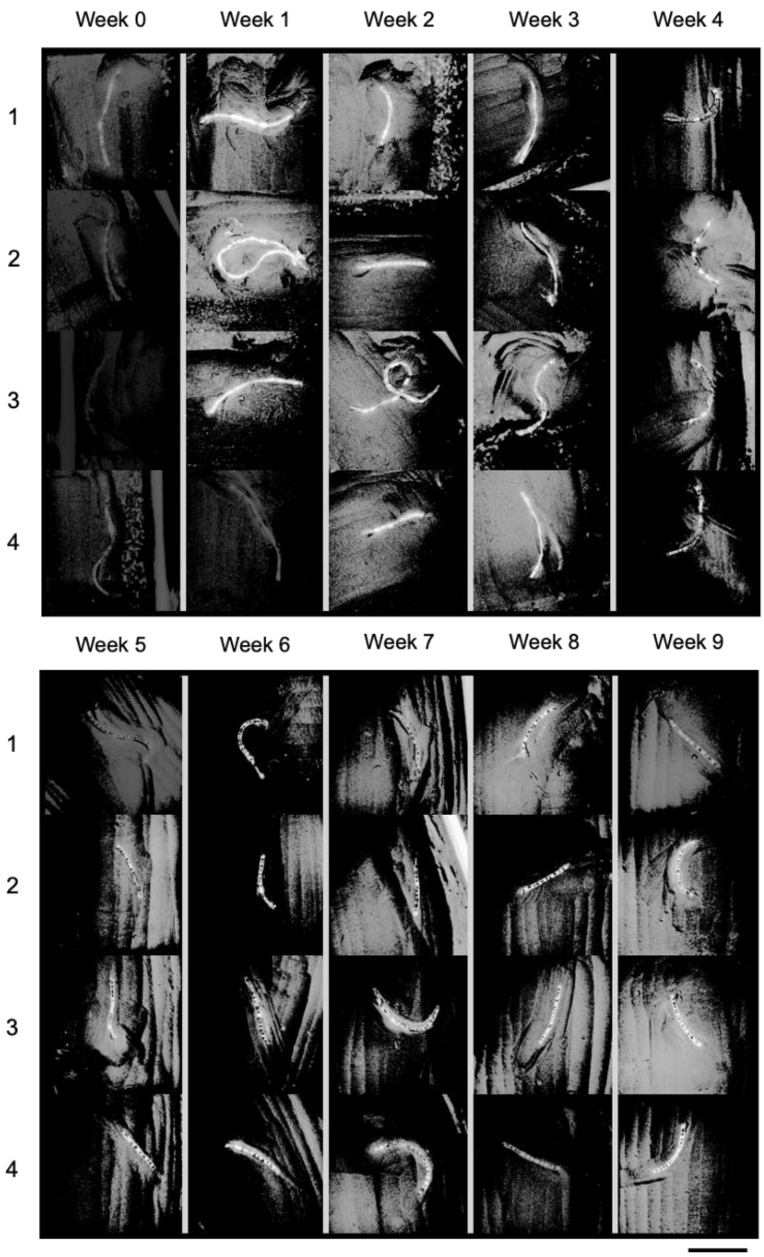

Figure S47: Morphological changes of the tubular macroalgae, *Chaetomorpha sp.*, over time, for all samples (1 to 4). Standardized grey scale images presented at a logarithmic scale obtained by multispectral macroimaging: illumination 365 nm, detection 650±30 nm. Scale bar: 1 cm.

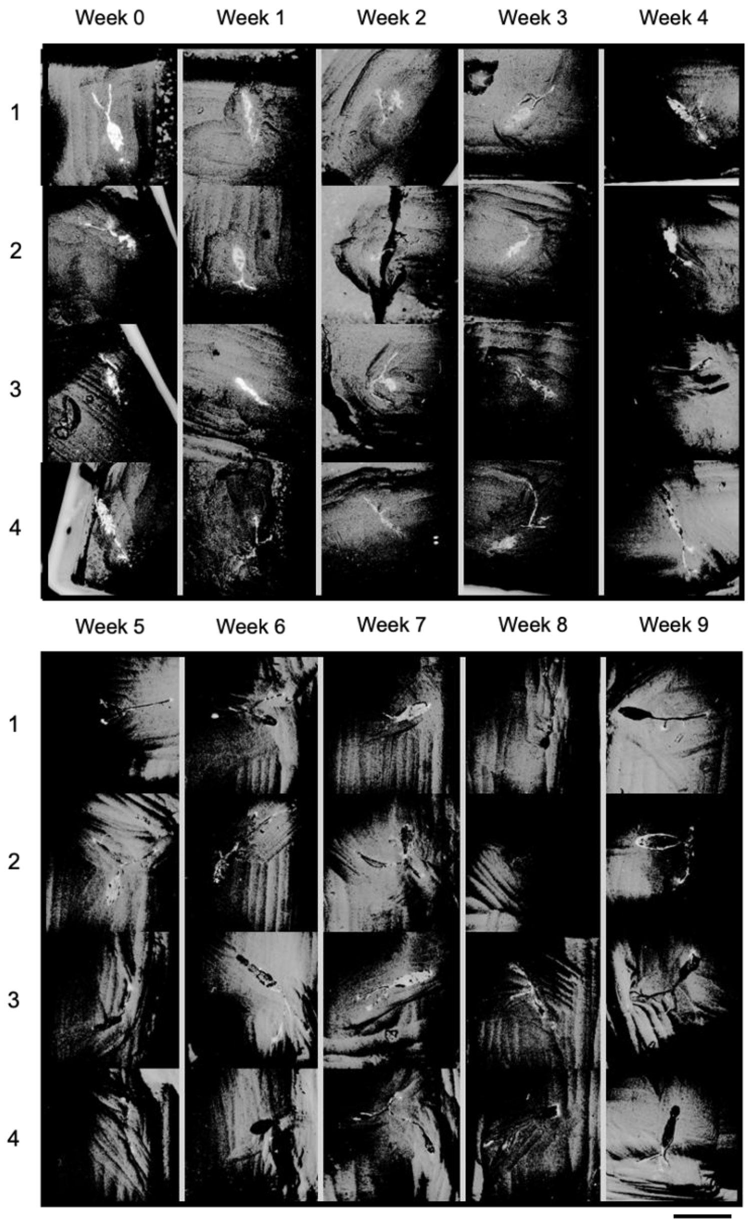

Figure S58: Morphological changes of the frondose macroalgae, *Caulerpa sp.*, over time, for all samples (1 to 4). Standardized grey scale images presented at a logarithmic scale obtained by multispectral imaging: illumination 365 nm, detection 650±30 nm. Scale bar: 1 cm.

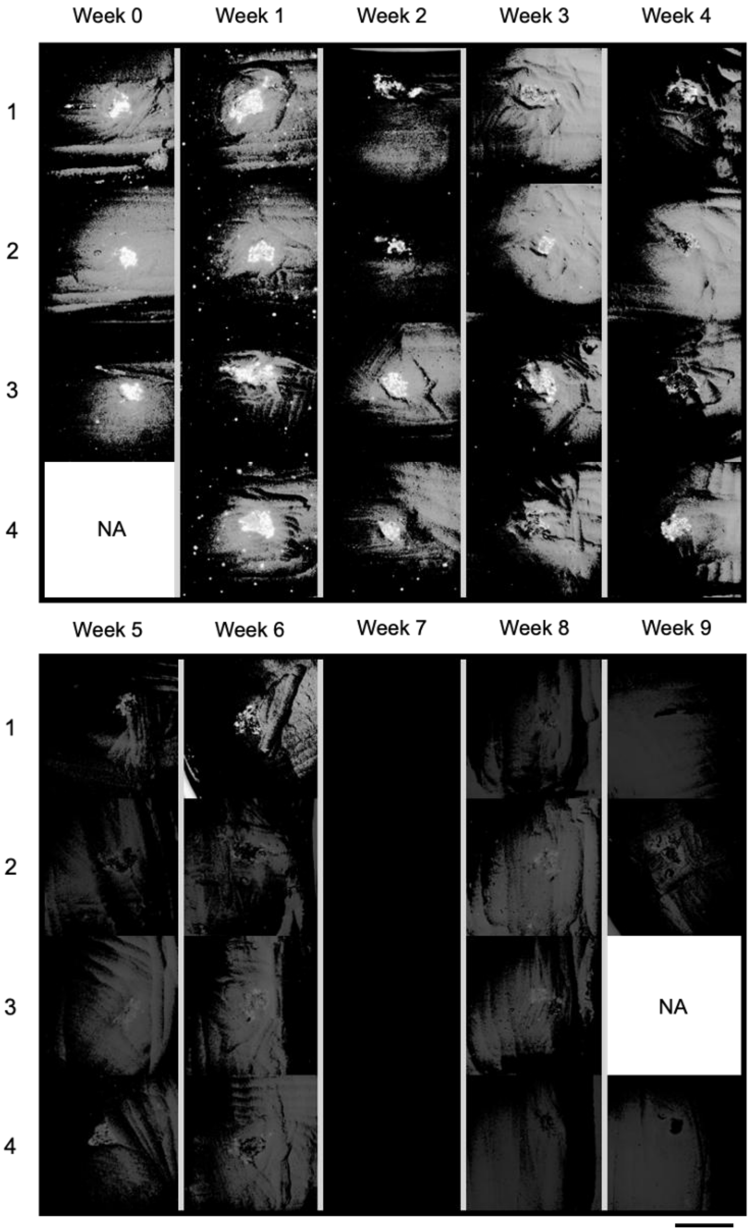

Figure S69: Morphological changes of the mat-forming green algae over time, for all samples (1 to 4). Standardized grey scale images presented at a logarithmic scale obtained by multispectral imaging: illumination 365 nm, detection 650±30 nm. Scale bar: 1 cm.

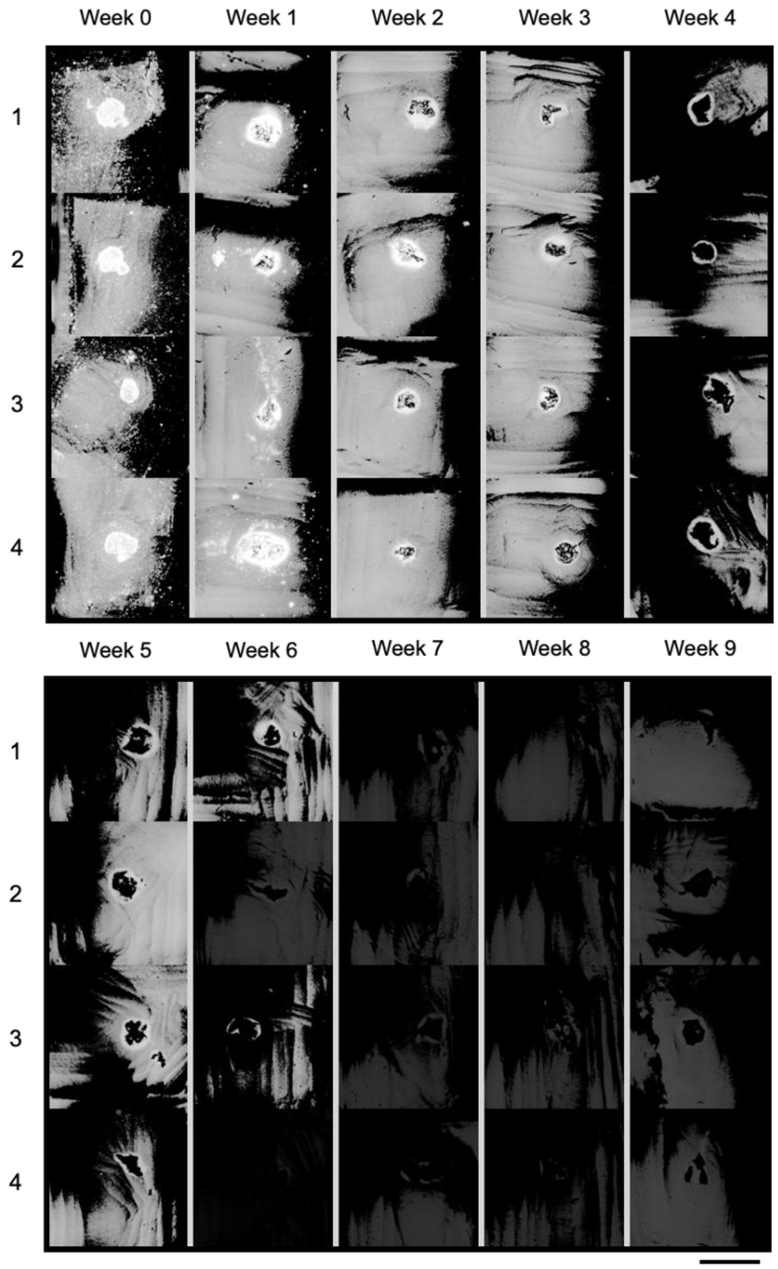

Figure S7~~10~~: Morphological changes of the red cyanobacteria over time, for all samples (1 to 4). Standardized grey scale images presented at a logarithmic scale obtained by multispectral imaging: illumination 525 nm, detection 650±30 nm. Scale bar: 1 cm.

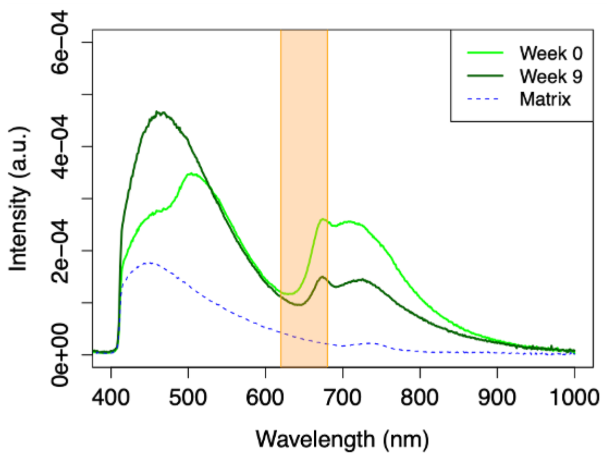

Figure S8: UV−vis−NIR spectroscopy of the tubular macroalgae, after 0 and 9 weeks of decay. Luminescence bands in the 650–800 nm domain are characteristic of chlorophyll. No chlorophyll luminescence is observed for the kaolinite sediment, with the luminescence centred around ~450 nm originating from cellulosic 'dust' residue on the sample surface (note that the band at ~730 nm is the second harmonic of the 365 nm illumination, visible here due to the extremely low overall luminescence signal produced by only a few 'dust' residues). The orange selection represents the 650 ±30nm detection filter used in this study.

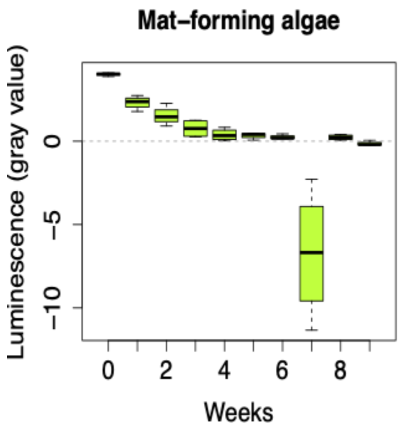

Figure S9: Standardized luminescence values of the mat-forming green algae, over time. Including the Week 7 data removed from the main manuscript, as it is considered an outlier. Illumination at 365 nm wavelength and luminescence detected with a 650±30 nm filter.

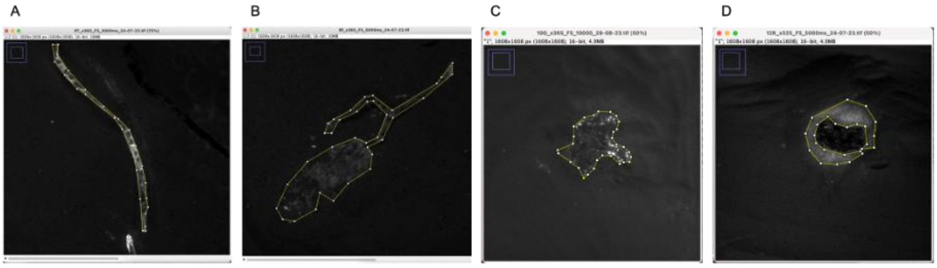

Figure S10: Examples of outline selection for each organism. (A) Tubular macroalga sample after 3 weeks of decay. Here the entire sample is shown outlined for measurement. (B) Frondose macroalga sample after 3 weeks of decay. Here the entire sample is shown outlined for measurement. In addition, both the frond and the stolon were outlined for separate measurements. (C) Mat-forming algae sample after 3 weeks of decay. Here the entire sample is shown outlined for measurement. (D) Red cyanobacteria sample after 3 weeks of decay. Here the luminescence halo is shown outlined for measurement. In addition, the sample alone was outlined for measurement.
